# The mediating role of relative harm perception in the association between social media marketing exposure-engagement patterns and e-cigarette use behaviors: A cross-sectional study

**DOI:** 10.18332/tid/219983

**Published:** 2026-05-14

**Authors:** Naeun Kang, Heewon Kang, Sung-il Cho, Susan Park

**Affiliations:** 1 Department of Public Health Sciences, Graduate School of Public Health, Seoul National University, Seoul, Republic of Korea; 2 Department of Health Administration, Daejin University, Gyeonggi, Republic of Korea; 3 Institute of Health and Environment, Seoul National University, Seoul, Republic of Korea; 4 College of Medicine, Inha University, Incheon, Republic of Korea

**Keywords:** electronic cigarettes, ends, social media, online marketing, Republic of Korea

## Abstract

**INTRODUCTION:**

Social media marketing is a key driver of e-cigarette use. While active engagement facilitates message internalization, research on how specific engagement types influence behaviors via relative harm perception remains limited. This study investigates the mediating role of relative harm perception in the relationship between social media marketing engagement and e-cigarette curiosity, current use, and quit attempts.

**METHODS:**

Data from a cross-sectional web-based survey of 3400 adults aged 19–69 years in the Republic of Korea were analyzed. Participants were recruited in February 2025 from an opt-in online panel using quota-controlled, non-probability sampling designed to balance sex and tobacco-use strata. Respondents were categorized into five groups based on the combination of marketing exposure frequency (high vs low) and behavioral engagement (active vs passive), including a no-exposure group. Weighted multivariable logistic regression and mediation analyses were conducted using a regression-based approach with the survey package in R.

**RESULTS:**

Active engagement was significantly associated with lower relative harm perception and increased e-cigarette use. In the ‘Low frequency-active engagement’ group, harm perception fully mediated the relationship between engagement and use. Conversely, the ‘High frequency-active engagement’ group demonstrated strong direct effects on use (adjusted odds ratio, AOR=7.23; 95% CI: 2.99–17.48) and curiosity (AOR=5.07; 95% CI: 1.80–14.27), bypassing mediation. In contrast, the ‘High frequency-passive engagement’ group exhibited an inverse association, showing lower curiosity and higher harm perception.

**CONCLUSIONS:**

Active engagement with e-cigarette-related marketing content on social media was associated with lower perceived relative harm and higher odds of e-cigarette curiosity and current use, whereas frequent passive exposure was associated with higher perceived harm and lower curiosity among non-users. These findings underscore the potential importance of incorporating engagement-based metrics when monitoring digital marketing exposure and when evaluating regulatory approaches.

## INTRODUCTION

Despite accumulating evidence regarding the health impacts of e-cigarettes or Electronic Nicotine Delivery Systems (ENDS), public perceptions of their harms remain mixed, and uncertainty-oriented messages can attenuate perceived risk^1,2^. In particular, the emerging trend of using e-cigarette as a delivery device for new illicit drugs, demonstrates that e-cigarettes can serve as a conduit for drug exposure among adolescents^3^, creating social repercussions that extend beyond direct health effects.

However, the gravity of these risks is often obscured by strategic industry communication that frames the devices as harmless or trendy lifestyle accessories. Research indicates that such marketing exposure tends to emphasize uncertainty regarding long-term health effects or obfuscate risks, thereby lowering consumers’ harm perception^1^. This distortion is particularly prevalent on social media, an interactive digital environment that tobacco companies are utilizing to maximize reach by facilitating direct interaction via hashtags (#) and amplifying effects through influencers^4^. Unlike traditional one-way delivery, social media enable two-way interaction (e.g. liking, commenting, sharing), which may amplify and reinforce marketing messages through user participation and engagement^5-7^.

Social Cognitive Theory (SCT) offers a relevant framework for this digital context, positing that human behavior is shaped through dynamic interactions among personal, behavioral, and environmental factors^8^. When applied to social media, this perspective implies that user interaction with content, rather than mere exposure, may play a key role in shaping attitudes and behaviors. Indeed, prior work suggests that active engagement (e.g. liking or clicking) may intensify message processing and internalization compared with passive viewing^5,6^. Epidemiological studies further indicate that engagement with tobacco-related social media content is associated with ENDS use and subsequent uptake^7,9-12^. Notably, a meta-analysis of over 139000 individuals confirmed that engagement with tobacco content is associated with a twofold increase in the odds of past 30-day use and susceptibility among never users^9^. Consistent with this, longitudinal studies including cross-lagged analyses have demonstrated that such engagement significantly elevated the risk of initiation and sustained use one-year later^10-12^.

Despite these clear indications of risk, recent studies still focus on metrics of passive exposure rather than the interactive nature of user behavior^13,14^. Furthermore, exposure and engagement are often examined as distinct predictors, leaving their combined, interactive effects on cognitive processing less understood. Recent evidence syntheses indicate that exposure to e-cigarette advertising, promotion, and sponsorship (APS) – particularly via digital and social media channels – is generally associated with more favorable cognitions, greater susceptibility or intention, and higher likelihood of use, although effect sizes and directions vary by channel and study design^4^. Therefore, to build upon these foundational insights, further research is needed to disentangle the specific mechanisms by which active engagement reshapes user perceptions. Existing literature indicates that lowered relative harm perception is one of the drivers of tobacco use behavior^15,16^. Specifically, it stimulates curiosity, defined as interest independent of usage intentions^17^, which serves as a key mediator between advertising exposure and trial^18,19^. Furthermore, the perception that e-cigarettes are less harmful than combustible cigarettes (CCs) reinforces continued use among existing users or may consolidate dual use^20^. However, while the relationship between general advertising exposure and ever e-cigarette use and susceptibility to e-cigarette use has been investigated^21^, the specific mechanisms linking active engagement to these outcomes have yet to be fully elucidated^22^. Thus, extending beyond binary metrics to capture the nuances of interaction could offer a more comprehensive understanding of the cognitive pathways leading to tobacco use.

Therefore, this study aims to investigate the association between e-cigarette marketing engagement and tobacco use behaviors by introducing the Exposure-Engagement Patterns to disentangle these interactive effects. Specifically, this study aims to empirically identify the mediating effect of relative harm perception of e-cigarettes relative to CCs on the relationship between marketing engagement and tobacco use behaviors.

## METHODS

### Study data

This study utilized an online survey from Macromill Embrain’s research panel (n=1774777 as of 1 February 2025), drawing upon their large opt-in research panel structured by sex, age, and geographical distribution. The data collection took place during 6–13 February 2025, and included adults aged 19–69 years residing across the Republic of Korea.

A quota-controlled sampling approach (non-probability) was used to meet prespecified targets by sex and tobacco-use strata to facilitate subgroup comparisons (1700 current tobacco product users and 1700 non-users, balanced by sex). Within the tobacco-user stratum, additional quotas were applied to enroll approximately equal numbers of current combustible cigarette smokers and current heated tobacco product and/or e-cigarette users.

The survey instrument comprehensively captured several domains, including sociodemographic characteristics (e.g. income, education level, and residence), current e-cigarette use, exposure frequency and engagement type to e-cigarette-related marketing on social media, and perceptions regarding the relative harm of e-cigarettes compared to CCs.

Recruitment was managed by the panel vendor using a quota-controlled system. Invitations remained open until the pre-specified quotas (by sex and tobacco-use strata) were met; once a quota was filled, the survey was automatically closed for that stratum and additional responses were no longer accepted. Therefore, the final sample size reflects the planned quota targets rather than an open-ended enrollment process. All participants provided electronic informed consent prior to participation, and the data were analyzed in de-identified form. This study was approved by the Institutional Review Board of Seoul National University (IRB No. 2412/004-015).

Prior to analysis, the de-identified dataset was screened for eligibility and completeness. Due to the forced-response setting of the online survey platform, there were no missing values for any of the key measures. The survey platform applied forced-response settings for administered items and automated completeness checks before submission; therefore, item nonresponse for key analytic variables was 0%. Items on exposure frequency and engagement were administered only to respondents reporting e-cigarette-related marketing exposure on social media in the past 30 days, so non-administration due to skip patterns was treated as structural inapplicability rather than missingness. The final analytic sample (n=3400) therefore reflects fully completed questionnaires that met all prespecified eligibility criteria and quota targets.

### Measures

*Social media marketing exposure-engagement patterns*

To comprehensively analyze the impact of social media marketing on e-cigarette, the Exposure-Engagement Patterns was constructed, which is a composite independent variable. This variable integrates two dimensions: exposure frequency and behavioral engagement. First, the exposure dimension was assessed using the question: ‘How often have you seen posts related to e-cigarette (e.g. devices, liquids) on social media in the past 30 days?’. Responses were dichotomized into ‘High frequency’ representing daily exposure and ‘Low frequency’ representing non-daily exposure. Second, the engagement dimension was measured by asking: ‘What actions did you take regarding e-cigarette contents seen on social media in the past 30 days?’. The respondents who selected any of ‘liked’, ‘commented’, and ‘clicked’ for details were categorized as the ‘Active engagement’ group, while those who selected ‘did nothing’ were categorized as the ‘Passive engagement’ group.

Finally, by combining these two dimensions, participants were classified into four mutually exclusive types, which consisted of: ‘High frequency-active engagement’, ‘Low frequency-active engagement’, ‘High frequency-passive engagement,’ and ‘Low frequency-passive engagement’. Respondents who did not report any e-cigarette-related marketing exposure on social media in the past 30 days – including those exposed only through other channels – were classified as ‘No exposure’ for the social-media-specific exposure–engagement variable and served as the reference category.

To assess whether exposure frequency and engagement capture related but nonredundant aspects of social media marketing contact, we computed the phi (φ) coefficient between the dichotomized exposure and engagement variables. The association was statistically significant but small (φ=0.112, p=0.014), suggesting that exposure frequency and engagement are related yet not interchangeable^23^.

The Exposure–Engagement Patterns variable represents an analytic typology derived from two observed dimensions – exposure to e-cigarette-related marketing content on social media and behavioral engagement with such content. This classification is grounded in prior literature that distinguishes exposure from engagement with tobacco-related content on social media and indicates that these are related but nonredundant forms of contact^7,9,11^. The survey items used to operationalize these constructs are provided in the Supplementary file.

*E-cigarette-related variables*

Three distinct outcomes were used in this study, based on e-cigarette use status. First, for the total sample (n=3400), ‘current users’ were defined as using e-cigarettes either daily or occasionally at the time of the survey, while ‘current non-users’ was defined as not currently e-cigarette users including former and never users. Second, among current users (n=571), ‘quit attempts’ were measured by whether they had attempted to stop using e-cigarettes in the past 12 months (yes vs no). Lastly, curiosity was assessed by asking current non-users (n=2829), ‘Have you ever been curious about e-cigarette?’ on a 5-point scale. Responses were dichotomized into ‘yes’ (‘very much’ to ‘neutral’) and no (‘Not really’ to ‘Not at all’).

*Harm perception of e-cigarettes relative to combustible cigarettes*

Relative harm perception was assessed using the question: ‘Compared to CCs, how harmful to health do you think e-cigarettes are?’. Respondents chose from five options from ‘Much more harmful’ to ‘Much less harmful,’ with ‘Equally harmful’ as the midpoint. For the analysis, these responses were dichotomized into two categories of ‘Same or more harmful’ and ‘Less harmful’.

*Potential covariates*

Sociodemographic characteristics were included as covariates: sex, age, residence, and socioeconomic status measured by monthly income and education level. Age was categorized into five groups (19–29, 30–39, 40–49, 50–59, and 60–69 years). Residence was dichotomized into the Capital area (Seoul, Incheon, and Gyeonggi) versus non-Capital area (all other regions). Monthly income (KRW) was categorized into three groups: Low (≤3000000), Middle (3000001–5000000), and High (≥5000001). Education level was divided into four categories, but collapsed into two categories, which consist of high school or lower (middle school or high school) and college or higher (college graduate or graduate/postgraduate education) for multivariable adjustment. These covariates were selected *a priori* as potential confounders of the association between social media marketing engagement and e-cigarette-related outcomes^21^.

*Statistical analysis*

**Figure 1:**
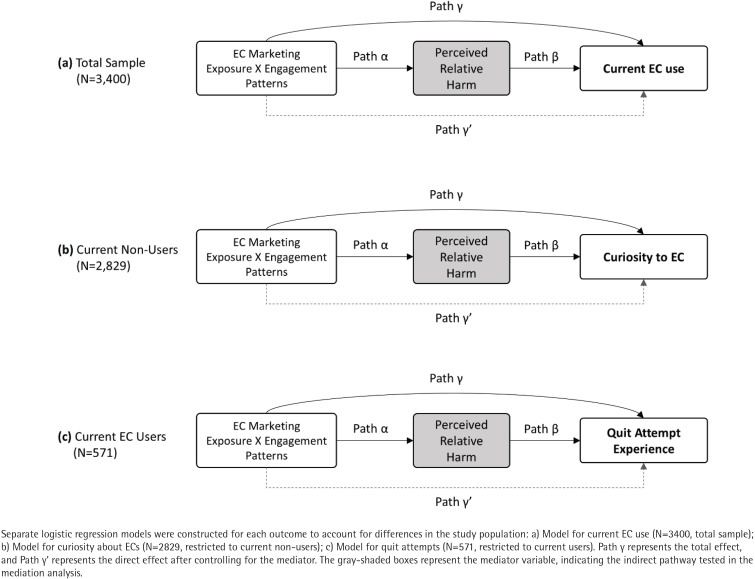
Path diagram illustrating the mediating role of EC harm perception compared with CC on the relationship between social media marketing on EC and EC current use, curiosity about EC, and quit attempts in a cross-sectional web-based survey of adults in the Republic of Korea, 6–13 February 2025 (N=3400)

For enhanced generalizability, survey weights were computed by considering prevalence estimates stratified by sex and current use status of three product types, using prevalence benchmarks derived from the 2019–2023 Korea National Health and Nutrition Examination Survey (KNHANES). Descriptive statistics were used to present the general characteristics of the study population. Differences in categorical variables were examined using the Rao-Scott chi-squared test to account for the complex sampling design. Drawing upon the mediation framework described by Jiang et al.^21^, mediation analyses were conducted using a regression-based approach with a series of weighted multivariable logistic regression models^21^. These analyses examined whether e-cigarette harm perception compared to CC (mediator) mediated the association between e-cigarette marketing engagement on social media and three outcomes to account for differences in the target population: current e-cigarette use (the full study sample), curiosity about e-cigarette (current non-users), and quit attempts (current users). Specifically, the current analysis estimated the association between marketing engagement type and e-cigarette harm perception (Path α; Figure 1), as well as the association between the mediator and each outcome while adjusting for the independent variable (Path β). Total effects (Path γ) and direct effects controlling for the mediator (Path γ') were also estimated. Given the cross-sectional design, mediation parameters are interpreted as associations consistent with the hypothesized pathway rather than evidence of temporality or causal mediation^24^. Under a causal interpretation, the regression-based mediation framework assumes correct model specification and no unmeasured confounding of the exposure–mediator and mediator–outcome relationships^25^. Such an interpretation further requires no unmeasured exposure–outcome confounding and no mediator–outcome confounder affected by the exposure^25^. All multivariable models adjusted for sex, age, residence, monthly income, and education level. The path diagram is illustrated in Figure 1.

The product-of-coefficients method was used to test the significance of the indirect effect, rather than the difference method. The difference method was avoided because of the rescaling of error variance, in non-linear models like logistic regression, rendering coefficients incomparable across models^25^. Consequently, the product method serves as a valid alternative for testing the null hypothesis of no mediation in this context^25^. For binary outcomes, indirect effects were exponentiated and presented as odds ratios with 95% confidence intervals.

All statistical analyses were performed using R software (version 4.5.1), with the *survey* package used to account for the complex sampling design and the *dplyr* package used for data management. Sampling weights were applied to all descriptive and inferential analyses. A two-sided p<0.05 was considered statistically significant.

## RESULTS

### General characteristics of the study population

A total of 3400 respondents were selected in the final analysis. Table 1 shows significant sociodemographic differences between current e-cigarette users and non-users in terms of sex and age (all p<0.05), with current users being predominantly male (82.3%) compared to non-users (52.9%). For residence (p=0.901), income (p=0.153), and education level (p=0.568), there were no significant differences between the two groups.

**Table 1:** Descriptive characteristics in a cross-sectional web-based survey of adults in the Republic of Korea, 6–13 February 2025 (N=3400)

*Variables*		*Total* *(N=3400)*	*Current EC use*		*p*
			*No (N=2829)*	*Yes (N=571)*	
**Sociodemographic characteristics**					
**Sex**	Male	1834 (53.9)	1503 (52.9)	331 (82.3)	<0.001
	Female	1566 (46.1)	1326 (47.1)	240 (17.7)	
**Age** (years)	19–29	462 (12.5)	322 (12.0)	140 (25.1)	<0.001
	30–39	617 (16.1)	474 (15.9)	143 (20.9)	
	40–49	710 (19.2)	567 (18.9)	143 (27.2)	
	50–59	826 (24.5)	738 (24.9)	88 (14.0)	
	60–69	785 (27.7)	728 (28.2)	57 (12.8)	
**Residence**	Capital area	1291 (39.9)	1089 (40.0)	202 (39.5)	0.901
	Non-Capital area	2109 (60.1)	1740 (60.0)	369 (60.5)	
**Income per month** (KRW)	>5000000	908 (26.0)	759 (26.0)	149 (26.5)	0.153
	3000000-5000000	1225 (34.9)	1003 (34.7)	222 (40.2)	
	<3000000	1267 (39.1)	1067 (39.4)	200 (33.3)	
**Education level**	<High school	20 (0.7)	16 (0.7)	4 (0.7)	0.568
	High school	724 (19.8)	591 (19.7)	133 (22.3)	
	College graduate	2281 (66.9)	1891 (66.9)	390 (66.7)	
	Graduate/postgraduate	375 (12.7)	331 (12.8)	44 (10.2)	
**Social media marketing**					
**Marketing exposure-engagement patterns**	No exposure	2916 (89.1)	2507 (89.5)	409 (77.9)	<0.001
	Low frequency-passive engagement	208 (5.8)	162 (5.7)	46 (7.9)	
	Low frequency-active engagement	142 (2.5)	94 (2.4)	48 (5.9)	
	High frequency-passive engagement	63 (1.7)	47 (1.7)	16 (1.4)	
	High frequency-active engagement	71 (0.9)	19 (0.7)	52 (6.9)	
**Relative harm perception on ECs relative to CCs**					
**Relative harm perception**	Same or more	2489 (76.2)	2154 (77.2)	335 (50.0)	<0.001
	Less harmful than CC	911 (23.8)	675 (22.8)	236 (50.0)	
**Curiosity about** ECs (N=2829)					
**Curiosity about ECs**	No	-	1597 (68.3)	-	-
	Yes	-	1232 (31.7)	-	
**EC quit attempts** (N=571)					
**EC quit attempts**	Yes	-	-	275 (46.5)	-
	No	-	-	296 (53.5)	

Values are presented as an unweighted number (n) and a weighted percentage (%). P-values were calculated using the Rao-Scott chi-squared test to account for the complex sampling design. EC: e-cigarette. CC: combustible cigarette. KRW: 1000 Korean Won about US$0.68.

In terms of social media marketing engagement (p<0.001), the majority of the respondents (89.1%) reported no exposure in the past 30 days. In contrast, the ‘High frequency-active engagement’ group was substantially more prevalent among current users (6.9%) compared to non-users (0.7%). Regarding relative harm perception (p<0.001), 77.2% of non-users viewed e-cigarettes as equally or more harmful than CCs, whereas both ‘less harmful’ and ‘same or more harmful’ perceptions among current users were each reported by 50.0% of the group. For e-cigarette-related behavioral outcomes, 31.7% (n=1232) of non-users expressed curiosity about e-cigarettes, while 46.5% (n=275) of current users reported having made a quit attempt in the past 12 months.

### Relative harm perception of e-cigarettes by marketing engagement

Table 2 presents the distribution of exposure–engagement patterns according to the perceived relative harm of e-cigarettes compared with CCs. The overall distribution differed significantly between harm-perception categories (p=0.003). Specifically, groups with active social media engagement accounted for a larger share of the ‘less harmful’ category compared to other harm-perception groups. For instance, the ‘High frequency-active engagement’ group represented 1.5% of the individuals who perceived e-cigarettes as less harmful, which was approximately double its share (0.7%) within the ‘same or more harmful’ category. Conversely, the ‘No exposure’ group accounted for a larger proportion of the ‘same or more harmful’ category (89.8%) than the ‘less harmful’ category (86.9%).

**Table 2:** Perceived relative harm of ECs by social media marketing exposure-engagement patterns in a cross-sectional web-based survey of adults in the Republic of Korea, 6–13 February 2025 (N=3400)

*Variables*		*Relative harm perception of ECs* *compared to CCs*		
		*Same or more* *(N=2489)*	*Less harmful* *(N=911)*	*p*
Marketing exposure-engagement patterns	No exposure	2160 (89.8)	756 (86.9)	0.003
	Low frequency-passive engagement	136 (5.5)	72 (6.9)	
	Low frequency-active engagement	94 (2.0)	48 (3.9)	
	High frequency-passive engagement	49 (2.0)	14 (0.8)	
	High frequency-active engagement	50 (0.7)	21 (1.5)	

Values are presented as an unweighted number (n) and a weighted percentage (%). P-value was calculated using the Rao-Scott chi-squared test to account for the complex sampling design. EC: e-cigarette. CC: combustible cigarette.

### E-cigarette curiosity and quit attempts by relative harm perception

Table 3 summarizes e-cigarette-related outcomes according to relative harm perception. Among current non-users, the perception of relative harm was significantly associated with curiosity (p<0.001). Specifically, among current non-users who were curious about e-cigarettes, 30.5% perceived e-cigarettes as less harmful than CCs, compared with 19.2% among those who were not curious. For current e-cigarette users, a significant association was found between harm perception and attempts to quit in the past 12 months (p<0.001). Among users who reported a quit attempt, 66.3% perceived e-cigarettes as equally or more harmful than CCs, compared with 35.8 % among those without a quit attempt.

**Table 3:** Curiosity and quit attempts of EC by perceived relative harm in a cross-sectional web-based survey of adults in the Republic of Korea, 6–13 February 2025 (Current EC non-users, N=2829; Current EC users, N=571)

*Variables*		*Curiosity about EC* *among current non-users* *(N=2829)*			*Quit attempts among current users* *(N=571)*		
		*No* *(N=1597)*	*Yes* *(N=1232)*	*p*	*No* *(N=296)*	*Yes* *(N=275)*	*p*
Relative harm perception of ECs compared to CCs	Same or more	1305 (80.8)	849 (69.5)	<0.001	150 (35.8)	185 (66.3)	<0.001
	Less harmful	292 (19.2)	383 (30.5)		146 (64.2)	90 (33.7)	

Values are presented as an unweighted number (n) and a weighted percentage (%). P-values were calculated using the Rao-Scott chi-squared test to account for the complex sampling design. EC: e-cigarette. CC: combustible cigarette.

### Mediation analysis of relative harm perception on e-cigarette outcomes

Figure 1 illustrates the mediation model and path definitions, and Table 4 presents the corresponding estimates. Perceiving e-cigarettes as less harmful than CCs was significantly associated with a higher likelihood of current use (Path β: adjusted odds ratio, AOR=2.72; 95% CI: 1.85–4.00). Regarding the marketing engagement types, the ‘Low frequency-active engagement’ group showed a significant indirect effect (AOR=1.83; 95% CI: 1.09–3.06). This indicates that active engagement, even at low frequency of marketing exposure, was associated with a higher probability of perceiving e-cigarettes as less harmful (Path α: AOR=1.82), which in turn increased the likelihood of current e-cigarette use. Conversely, the ‘High frequency-active engagement’ group demonstrated a substantial direct effect (AOR=7.23; 95% CI: 2.99–17.48) on current use that was not mediated by harm perception, resulting in the highest total effect (AOR=7.98; 95% CI: 3.37–18.89) among all groups. The ‘High frequency-passive engagement’ group was less likely to perceive e-cigarettes as less harmful (Path α: AOR=0.37; 95% CI: 0.16–0.88), leading to a significant protective indirect effect (AOR=0.37; 95% CI: 0.15–0.95).

**Table 4:** Mediating role of EC harm perception in the associations of social media marketing exposure-engagement patterns with current EC use, quit attempts, and curiosity about EC in a cross-sectional web-based survey of adults in the Republic of Korea, 6–13 February 2025 (N=3400)

	*Path α* *AOR (95% CI)*	*Path β* *AOR (95% CI)*	*Indirect effect* *AOR (95% CI)*	*Direct effect* *AOR (95% CI)*	*Total effect* *AOR (95% CI)*
**Current EC use (N=3400)**					
No exposure (ref.)					
Low frequency-passive engagement	1.02 (0.74–1.43)	**2.72 (1.85–4.00)**	1.02 (0.73–1.43)	1.29 (0.64–2.60)	1.23 (0.61–2.46)
Low frequency-active engagement	**1.82 (1.15–2.90)**	**2.72 (1.85–4.00)**	**1.83 (1.09–3.06)**	2.03 (0.89–4.65)	**2.28 (1.01–5.15)**
High frequency-passive engagement	**0.37 (0.16–0.88)**	**2.72 (1.85–4.00)**	**0.37 (0.15–0.95)**	0.86 (0.18–4.08)	0.73 (0.16–3.43)
High frequency-active engagement	2.00 (0.94–4.26)	**2.72 (1.85–4.00)**	2.00 (0.90–4.47)	**7.23 (2.99–17.48)**	**7.98 (3.37-18.89)**
**EC quit attempts** (N=571)					
No exposure (ref.)					
Low frequency-passive engagement	2.28 (0.53-9.76)	**0.28 (0.13–0.60)**	0.35 (0.05–2.48)	0.75 (0.17–3.28)	0.60 (0.15–2.46)
Low frequency-active engagement	0.68 (0.14–3.35)	**0.28 (0.13–0.60)**	1.63 (0.21–12.68)	1.42 (0.28–7.32)	1.56 (0.33–7.50)
High frequency-passive engagement	2.30 (0.09–60.73)	**0.28 (0.13–0.60)**	0.35 (0.01–23.70)	0.74 (0.02–23.23)	0.61 (0.02–15.58)
High frequency-active engagement	0.59 (0.14–2.56)	**0.28 (0.13–0.60)**	1.95 (0.29–13.13)	1.06 (0.24–4.70)	1.23 (0.29–5.12)
**Curiosity about EC** (N=2829)					
No exposure (ref.)					
Low frequency-passive engagement	0.97 (0.68–1.37)	**1.83 (1.52–2.21)**	0.98 (0.79–1.21)	1.15 (0.83–1.60)	1.14 (0.82–1.58)
Low frequency-active engagement	**1.93 (1.19–3.14)**	**1.83 (1.52–2.21)**	**1.49 (1.08–2.05)**	**11.66 (6.16–22.09)**	**12.21 (6.47–23.05)**
High frequency-passive engagement	**0.32 (0.13–0.83)**	**1.83 (1.52–2.21)**	**0.51 (0.28–0.93)**	0.88 (0.48–1.6)	0.81 (0.45–1.47)
High frequency-active engagement	2.42 (0.99–5.91)	**1.83 (1.52–2.21)**	1.71 (0.97–3.00)	**5.07 (1.8–14.27)**	**5.49 (1.97–15.32)**

Significant estimates (p<0.05) are bolded. EC: e-cigarette. CC: combustible cigarette. AOR: adjusted odds ratio. All models were adjusted for sociodemographic covariates, including sex, age, residence, monthly income, and education level. Indirect effects were calculated using the product-of-coefficients method. The coefficients for indirect effects represent the exponentiated effect mediated through relative harm perception.

In the analysis of quit attempts among current e-cigarette users, perceiving e-cigarettes as less harmful was significantly associated with lower odds of attempting to quit e-cigarette use in the past 12 months (Path β: AOR=0.28; 95% CI: 0.13–0.60). However, no statistically significant effects of social media marketing engagement types on quit attempts were observed in any exposure group.

For e-cigarette curiosity, a lower relative harm perception was significantly associated with increased curiosity (Path β: AOR=1.83; 95% CI: 1.52–2.21). A partial mediation effect was observed in the ‘Low frequency-active engagement’ group, showing both a significant indirect effect via harm perception (AOR=1.49; 95% CI: 1.08–2.05) and a remarkably strong direct effect (AOR=11.66; 95% CI: 6.16–22.09), culminating in a total effect of 12.21 (95% CI: 6.47–23.05). Similarly, the ‘High frequency-active engagement’ group exhibited a significant direct effect on curiosity (AOR=5.07; 95% CI: 1.80–14.27), although the indirect path was not statistically significant. Consistent with the findings for current use, the ‘High frequency-passive engagement’ group showed a reduced likelihood of perceiving e-cigarettes as less harmful, resulting in a lower indirect effect on curiosity (AOR=0.51; 95% CI: 0.28–0.93).

## DISCUSSION

This study provides a broad analysis of how social media marketing exposure and engagement were associated with the entire spectrum of e-cigarette-related behaviors, ranging from curiosity among non-users to current use and quit attempts among established users. The findings revealed that active engagement, rather than mere exposure frequency, serves as an associated factor of these outcomes, with its influence operating through both perception-mediated and direct pathways depending on the intensity of exposure.

Current mediation analysis demonstrated that people who actively responded (e.g. liking, clicking) were significantly more likely to perceive e-cigarettes as less harmful than CCs, which subsequently increased their likelihood of current use and curiosity. This empirically supports the SCT, implying that the interactive nature of social media facilitates the internalization of marketing messages (i.e. ‘e-cigarettes are safe’), which may contribute to turning passive audiences into susceptible potential users or to reinforcing current use. This interpretation is consistent with prior research showing that social cues and interactive features of e-cigarette-related content are associated with more favorable perceptions among viewers^26^.

Another finding was the inverse association observed in the ‘High frequency-passive engagement’ group among non-current users. Those exposed to marketing daily but who did not actively engage tended to perceive e-cigarettes as more harmful, which in turn suppressed their curiosity. This counter-intuitive result can be interpreted as a ‘backfire effect’ or advertising fatigue^27^, where excessive exposure triggers psychological reactance^28^. However, given the cross-sectional nature of this study, a reverse causality explanation is also plausible^24^. That means, individuals with pre-existing negative perceptions of e-cigarettes may knowingly choose not to engage with marketing content despite frequent exposure. This suggests that high harm perception might act as a filter, leading to passive behavior.

Recent evidence syntheses suggest that such counterintuitive or heterogeneous patterns are plausible in the broader literature. A systematic review of e-cigarette advertising, promotion, and sponsorship (APS) reported that APS exposure – particularly via digital/social media and retail environments – was generally associated with more favorable cognitions and higher likelihood of e-cigarette use, but findings for harm perceptions were mixed and associations varied by channel and study design. In that review, some analyses found no significant association for social media exposure with perceived harm, while certain traditional-media exposures were associated with higher perceived harm in adjusted models^4^. Taken together, these mixed findings support the interpretation that repeated exposure without engagement may not be uniformly persuasive and may reflect low receptivity or fatigue/reactance among individuals already skeptical of e-cigarette marketing^27-29^. In this context, distinguishing exposure frequency from behavioral interaction, as in the Exposure–Engagement Patterns, may help reconcile prior inconsistencies by separating frequently exposed but disengaged individuals from those who actively process and endorse marketing cues.

In contrast, regarding quit attempts among current users, neither exposure frequency nor engagement type showed a statistically significant impact. This null result suggests that external marketing stimuli may have a limited impact on established users, whose behaviors might be more resistant to change due to potential dependence or established habits^29^. While marketing effectively lowers the barrier to entry for non-users, it appears less effective in altering the cessation trajectory of those who have already formed a habit loop.

In addition to these external factors, for quit attempts among current e-cigarette users, perceiving e-cigarettes as less harmful than CCs was associated with lower odds of reporting a quit attempt in the past 12 months. This pattern is consistent with the interpretation that lower perceived risk may reduce motivation to discontinue e-cigarette use, whereas higher perceived harm may act as a trigger for cessation attempts^20,22^. Given the cross-sectional design, reverse causality and residual confounding remain plausible – for example, users who recently attempted to quit may report higher harm perceptions, and unmeasured factors such as nicotine dependence and cessation-related cognitions may influence both harm perception and cessation behavior^24,25,29^.

Taken together, while causal inference remains limited by the cross-sectional design^24^, these findings suggest that engagement-based indicators may be useful for digital marketing surveillance and for prioritizing regulatory evaluation^7,9,30,31^. For non-users, in particular, the reach and interactive features of e-cigarette marketing on social media may merit particular attention in prevention-oriented regulatory evaluation, given their associations with harm perception and curiosity in this study^7,9,26^.

### Limitations

This study is subject to several limitations. First, the cross-sectional design precludes causal inference. While our mediation model posits that marketing engagement influences harm perception and subsequent behaviors based on SCT, it is equally possible that users’ pre-existing beliefs and usage status drive their engagement patterns on social media. Second, self-reported data introduce potential recall and social desirability biases. Additionally, the study relied on an opt-in online panel with quota controls. Although weighting was applied, the sample should not be interpreted as probability-based, and selection bias may limit generalizability. Third, since quit attempts were assessed only among current users, successful quitters were excluded, capturing only unsuccessful efforts. Fourth, this study analyzed social media as a composite environment and therefore could not examine platform-specific dynamics or content features that may shape engagement differently across platforms. Fifth, the dichotomization of continuous variables, specifically curiosity and harm perception, may have resulted in information loss, misclassification bias, and reduced statistical sensitivity to subtle nuances. Finally, the findings from South Korean adults may not generalize to adolescents or to settings with different regulatory and cultural contexts. Cultural backgrounds regarding tobacco control and digital literacy may vary across populations. Residual confounding is also possible, as unmeasured factors – particularly peer/social influences and dependence-related characteristics – may shape both engagement with marketing content and e-cigarette-related outcomes^2,4,19,29^. Future research should: 1) test temporal ordering using longitudinal designs with repeated measures of exposure, engagement, harm perceptions, and e-cigarette-related outcomes; 2) incorporate more objective indicators of digital marketing exposure and engagement (e.g. platform ad libraries or digital trace measures) alongside self-report; 3) examine platform-specific mechanisms and content characteristics that may differentially shape harm perceptions; and 4) evaluate policy interventions targeting interactive marketing features using quasi-experimental or natural-experiment approaches.

## CONCLUSIONS

This study uniquely integrates non-users and current e-cigarette users into a single analytical framework to comprehensively examine curiosity, current use, and quit attempts. Critically, by moving beyond the binary metric of exposure and incorporating engagement, this study offers a more nuanced and realistic analysis of social media influence. The findings suggest that behavioral engagement with e-cigarette-related marketing may be a more informative marker than exposure frequency alone. The unexpected discovery related to passive exposure, potentially due to skepticism or fatigue, is a significant contribution that warrants further exploration. Future research should investigate how marketing content interacts with exposure frequency to shape these outcomes.

## Supplementary Material



## Data Availability

The data supporting this research cannot be made available for privacy reasons. The data used in this study are not publicly available due to ethical restrictions. Access to the data is limited to researchers approved by the Institutional Review Board (IRB), and any secondary use of the data requires separate IRB approval.
