# Supplementary material for: The mediating role of relative harm perception in the association between social media marketing exposure-engagement patterns and e-cigarette use behaviors: A cross-sectional study
Source: Tob Induc Dis. 2026 May 14;24:10.18332/tid/219983. doi: 10.18332/tid/219983 (PMC13175034; doi:10.18332/tid/219983)
Supplement: Supplementary file 1 [file TID-24-60-s1.pdf]

Supplementary file:

Survey items (English translation)

A. Sociodemographic characteristics

Items	Response options
Sex	Male<br><br>Female
Age group	19-29<br><br>30-39<br><br>40-49<br><br>50-59<br><br>60-69
Current region of residence	Seoul<br><br>Busan<br><br>Daegu<br><br>Incheon<br><br>Gwangju<br><br>Daejeon<br><br>Ulsan<br><br>Gyeonggi<br><br>Gangwon<br><br>Chungbuk<br><br>Chungnam<br><br>Jeonbuk<br><br>Jeonnam

	Gyeongbuk<br>Gyeongnam<br>Jeju<br>Sejong
Highest level of education completed.	Middle school graduate<br>High school graduate<br>College graduate<br>Graduate school or higher
Average monthly income (KRW)	≤ 1,000,000 KRW<br>1,000,001–3,000,000 KRW<br>3,000,001–5,000,000 KRW<br>5,000,001–7,000,000 KRW<br>≥ 7,000,000 KRW

B. E-cigarette use, EC-related perceptions and behaviors

Items	Response options
Have you ever used liquid-based e-cigarettes, or do you currently use them?	Daily<br>Occasionally<br>Used in the past (former use)<br>Never used
In the past 12 months, have you ever stopped using liquid-based e-cigarettes for at least one day (24 hours) in an attempt to quit?	Yes<br>No
Have you ever been curious about liquid-based e-cigarettes?	Very much<br>Somewhat

	Neutral<br>Not really<br>Not at all
Compared with combustible cigarettes (regular cigarettes), how harmful to health do you think liquid-based e-cigarettes are?	Much more harmful<br>Somewhat more harmful<br>About the same<br>Somewhat less harmful<br>Much less harmful

C. Marketing exposure and engagement (in past 30 days)

Items	Response options
In the past 30 days, where have you seen advertisements for EC devices or e-liquids?	I have not seen any advertisements in the past 30 days<br>Convenience store or other retail outlets<br>Social media (e.g., Facebook, Instagram, X)<br>Newspapers or magazines<br>TV<br>Fairs/exhibitions, festivals, sports events, etc.<br>Clubs, internet cafés (PC rooms), restaurants, or cafés<br>Email messages<br>Outdoors/on the street<br>Other online websites

	Messaging apps (e.g., KakaoTalk open chat)<br><br>Other (please specify): ________
In the past 30 days, how often have you seen posts related to EC devices or e-liquids on social media?	Several times a day<br><br>About once a day<br><br>Several times a week<br><br>About once a week<br><br>Less than three times a month
What actions did you take regarding EC contents seen on social media in the past 30 days? (Select all that apply.)	I clicked "Like."<br><br>I left a comment<br><br>I clicked to view more details<br><br>I did nothing

Note: The survey was administered in Korean. The items above are an English translation of the questionnaire content used in this study. EC refers to liquid-based electronic cigarettes (vapes), and CC refers to combustible cigarettes.

Screening and skip patterns: Items on exposure frequency and engagement were administered only to respondents who reported any EC-related marketing exposure on social media in the past 30 days; respondents who did not report any EC-related marketing exposure on social media in the past 30 days—including those exposed only through other channels—were classified as 'No Exposure' for the social-media-specific exposure–engagement variable.

© 2026 Kang N. et al.
